# Dual and Triple Gut Peptide Agonists on the Horizon for the Treatment of Type 2 Diabetes and Obesity. An Overview of Preclinical and Clinical Data

**DOI:** 10.1007/s13679-025-00623-1

**Published:** 2025-04-11

**Authors:** Ioanna Α. Anastasiou, Georgia Argyrakopoulou, Maria Dalamaga, Alexander Kokkinos

**Affiliations:** 1 Diabetes Center, First Department of Propaedeutic Internal Medicine, Medical School, Laiko General Hospital, National and Kapodistrian University of Athens, 11527 Athens, Greece; 2 Department of Pharmacology, National and Kapodistrian University of Athens, 11527 Athens, Greece; 3 Diabetes and Obesity Unit, Athens Medical Center, 15125 Athens, Greece; 4 Department of Biological Chemistry, National and Kapodistrian University of Athens, 11527 Athens, Greece

**Keywords:** Glucagon, Glucagon-like peptide-1 receptor agonist, Glucose-dependent insulinotropic peptide receptor, Incretin, Obesity, Type 2 diabetes mellitus

## Abstract

**Purpose of Review:**

The development of long-acting incretin receptor agonists represents a significant advance in the fight against the concurrent epidemics of type 2 diabetes mellitus (T2DM) and obesity. The aim of the present review is to examine the cellular processes underlying the actions of these new, highly significant classes of peptide receptor agonists. We further explore the potential actions of multi-agonist drugs as well as the mechanisms through which gut-brain communication can be used to achieve long-term weight loss without negative side effects.

**Recent Findings:**

Several unimolecular dual-receptor agonists have shown promising clinical efficacy studies when used alone or in conjunction with approved glucose-lowering medications. We also describe the development of incretin-based pharmacotherapy, starting with exendin- 4 and ending with the identification of multi-incretin hormone receptor agonists, which appear to be the next major step in the fight against T2DM and obesity. We discuss the multi-agonists currently in clinical trials and how each new generation of these drugs improves their effectiveness. Since most glucose-dependent insulinotropic polypeptide (GIP) receptor: glucagon-like peptide- 1 receptor (GLP- 1) receptor: glucagon receptor triagonists compete in efficacy with bariatric surgery, the success of these agents in preclinical models and clinical trials suggests a bright future for multi-agonists in the treatment of metabolic diseases. To fully understand how these treatments affect body weight, further research is needed.

## Introduction

Type 2 diabetes mellitus (T2DM) and obesity are becoming more and more common worldwide, a phenomenon which greatly increases morbidity and mortality from conditions like cancer and heart disease, among many others [1]. The prevalence of T2DM is predicted to rise by 46% globally by 2045, suggesting that this issue becomes even more ominous as time progresses. A significant contributing factor to the prevalence of T2DM is obesity, which is strongly associated with serious consequences such as chronic kidney disease, hypertension, stroke, and liver disease that impair both the quality and quantity of life as life expectancy declines with rising body mass index (BMI) [2]. It is anticipated that between 2010 and 2030, the global prevalence of obesity will rise from 11 to 18% [1].

T2DM and obesity are two epidemics that seriously endanger human health. Weight loss of at least 5–10% reduces the severity of these disorders and their complications. Unfortunately, many people living with obesity cannot lose weight over the long term with lifestyle changes (diet and physical activity) alone [3]. Historically, anti-obesity drugs have shown a lot of negative side effects and only moderate efficacy. In addition to providing long-term weight loss and possibly remission of T2DM, bariatric surgery is not feasible for large populations, has specific indications, and may result in surgical complications as well as other issues like malabsorption and micronutrient deficiencies. The discovery of glucagon-like peptide 1 (GLP- 1) analogs pointed to the significant promise of gut hormones in the management of T2DM and obesity. Liraglutide and Semaglutide, a weekly GLP- 1 receptor agonist, was finally approved for both indications. It helps most patients control their T2DM and causes clinically significant weight loss. Polyagonist medications that stimulate several gut-brain pathways have the potential to revolutionize the treatment of T2DM and obesity. Tirzepatide is the first dual glucose-dependent insulinotropic polypeptide (GIP)/GLP- 1 receptor co-agonist approved for the treatment of T2DM in the USA, Europe, and the United Arab Emirates [4]; it causes an average weight loss of an astounding 22% in individuals with obesity. The development of gut hormone dual- and triagonists holds promise for treating obesity and T2DM with a level of effectiveness comparable to bariatric surgery. Research and development of safe and effective anti-obesity pharmacotherapies is urgently needed considering the current obesity epidemic. Lifestyle changes are important, but they frequently fall short of long-term weight management and glycemic control [5]. There is a paucity in the literature of papers addressing molecular mechanisms of new peptide polyagonists and extending these to preclinical and clinical data. This overview starts at the cellular level and extends to the human organism by examining the cellular mechanisms that influence the effects of these new and extremely important peptide receptor agonists. In addition, preclinical and clinical data were used to study the potential effects of drugs with multiple agonists and pathways through which gut-brain signaling pathways can be used to promote sustained weight loss without side effects. Finally, a comparative analysis involving bariatric surgery is carried out and costs are assessed.

## Receptors: Established Cell and Whole and Tissue Expression

### Glucagon‑like Peptide‑1 (GLP- 1) and Receptors

After consuming a meal, the L cells of the intestine produce and release the 30 amino acid peptide GLP- 1, which in turn promotes insulin secretion [6]. GLP- 1-based therapy exerts its pharmacological effects by activating the GLP- 1 receptor, a class of G protein-coupled receptor consisting of 463 amino acids. The receptor is a glycoprotein that, like all G protein-coupled receptors, has a seven-transmembrane helix domain and an N-terminal extracellular signal peptide [7]. When GLP- 1 receptors are activated, adenosine triphosphate (ATP) is converted to cyclic adenosine monophosphate (cAMP). Increased signaling through exchange proteins directly activated by cAMP (EPACs), specifically EPAC2, and activation of protein kinase A (PKA) follow the rise in cAMP. An increase in PKA activity also causes ATP-sensitive potassium channels to close, thereby depolarizing the cell membrane and opening voltage-gated calcium channels. This absorbs calcium and stimulates exocytosis of secretory granules, which in turn causes the release of insulin. Furthermore, the endoplasmic reticulum releases calcium in response to EPAC2 activation, increasing intracellular calcium levels and promoting exocytosis. Activation of the GLP- 1 receptor also prevents beta cell apoptosis. Additional consequences of GLP- 1 receptor activation include appetite suppression, cardioprotective effects, and inhibition of glucagon secretion associated with inhibition of gastric emptying [6]. Whether GLP- 1 increases energy expenditure has also been studied. Although there are data in animal models supporting that this is true, particularly in diet-induced obese mice, in which brown adipose tissue activity is regulated [8], there is no convincing evidence that this occurs in humans [6]. As a result, GLP- 1's primary effects include decreasing body weight, protecting against cardiovascular disease, and lowering fasting and postprandial blood glucose levels—all of which are therapeutic targets in T2DM. The short half-life of native GLP- 1-based therapy in the circulation (approximately two minutes after intravenous administration and one to five hours after subcutaneous administration) posed a problem during its early development [9]. For this reason, the first experiments of the glucose-lowering effect of GLP- 1 used continuous intravenous administration or intramuscular injections [10]. The short duration of action needed to be addressed to exploit the promising effects of GLP- 1 for the development of a clinically beneficial drug.

### Glucose-dependent Insulinotropic Polypeptide (GIP) Receptor

During fasting, K cells (found in the duodenum and jejunum) constitutively secrete the 42 amino acid protein GIP, which exhibits a marked increase in secretion after meals [11–13]. GIP was initially discovered because it inhibited the secretion of gastric acid. Since then, numerous investigations have revealed the effects of GIP on multiple organs [14]. The GIP receptor, a class of B G-protein-coupled receptors (GPCRs) that is widely expressed in a variety of tissues, is the binding site that mediates the systemic effects. The stomach, adipose tissue, bone, and pancreas all express GIP receptors, and these have also been found in the cerebral cortex, hippocampus, and olfactory bulb, among other parts of the brain [15, 16]. GIP is an incretin hormone that indirectly regulates glucagon secretion and increases glucose-stimulated insulin secretion [17]. GIP and GLP- 1 have opposing effects on glucagon release, despite their similarities. Studies conducted in isolated rat islets have shown that GIP does, in fact, increase intracellular cAMP levels, which in turn stimulate glucagon secretion [18]. The clinical application of GIP agonists for the treatment of diabetes is hampered by this increase in glucagon secretion, which has also been verified in both healthy and subjects with T2DM [19, 20]. In addition to its effects on insulin, GIP plays a crucial role in fat accumulation by promoting the activity of adipocyte-expressed lipoprotein lipase and promoting bone formation by preventing osteoclast apoptosis, as evidenced by the thinner bone trabeculae seen in mice lacking GIP receptors [20–22]. Better memory was also demonstrated by GIP-transgenic mice, which is most likely connected to increased neurogenesis [23]. In support to this theory, it has been demonstrated that GIP infusion increases the proliferation of neuronal progenitors in the dentate gyrus, while mice lacking GIP displayed deterioration in memorial tasks as a result of reduced neurogenesis [24].

### Glucagon Receptor

The first description of the hyperglycemic effects of glucagon was made more than a century ago [25, 26]. By attaching itself to its receptors, this hormone works in tandem with insulin to control blood sugar levels [14]. Glucagon's capacity to regulate hepatic glucose metabolism is primarily responsible for its hyperglycemic effects [27]. In order to maintain a steady supply of glucose, glucagon strongly stimulates glycogenolysis and suppresses glycogenesis in the liver [28, 29]. Many studies have revealed numerous additional functions of glucagon in the brain, liver, heart, and adipose tissue in addition to its well-known function in regulating blood sugar levels [30–32]. Nearly 40 years ago, it was discovered that the rat brain contained glucagon receptors, indicating that the hormone may play a part in controlling brain activity [33]. Accordingly, glucagon has been shown to reduce appetite, food intake, and promote weight loss in both humans and rodents [34, 35]. Despite the fact that the relationship between glucagon and body weight is well established, some research found no changes in food intake following glucagon administration, which suggests that appetite-independent mechanisms may play a role in the glucagon-mediated regulation of body weight [14]. Rats that receive a single subcutaneous dose of glucagon exhibit a sharp rise in metabolic rate, which is consistent with this theory [36]. By promoting oxygen consumption in brown adipose tissue (BAT), glucagon increases metabolic rate. This has been demonstrated by elevated BAT temperature in rats [37, 38]. By preventing lipogenesis and promoting lipolysis, glucagon also has lipolytic effects on white adipose tissue [14]. Hormone-sensitive lipase (HSL) in adipocytes is activated to produce the lipolytic effect, which is then enhanced by indirect processes such as secretion of growth hormone, cortisol, and adrenaline [39]. Adipose tissue is not the only area where glucagon affects lipid metabolism; the liver also experiences an increase in ketogenesis [40]. In fact, by continuously depriving the liver of esterified fatty acids and inhibiting the hepatic glycolytic pathway, glucagon promotes the production of ketone bodies [41–43] Hepatic ketogenesis is boosted as a result, and mitochondrial fatty acid oxidation is improved [14, 43]. By attaching itself to its receptors and activating adenylate cyclase (AC), which raises cAMP levels in the myocardium, glucagon also increases cardiac output. Glucagon has very rapid chronotropic and inotropic effects, peaking five minutes after administration and lasting for twenty minutes [14, 32].

### Pathways for Intracellular Signaling in the Langerhans Islets

GLP- 1 receptor, GIP receptor, and glucagon receptor are mainly expressed on beta, alpha, and delta cells in the islets of Langerhans, where they influence hormone secretion and regulate the survival and proliferation of endocrine cells. The search for GLP- 1 receptor agonists to treat T2DM was sparked by studies showing the insulinotropic and glucose-lowering effects of GLP- 1 in beta cells, as well as the hyperglycemic effects of GLP- 1 receptor antagonism [44]. Importantly, individuals with T2DM continue to benefit from GLP- 1's glucose-lowering effects. Insulin gene transcription and translation, as well as the enhancement of glucose-stimulated insulin secretion (insulinotropic effects), are caused by GLP- 1 receptor activation in the beta cell. GLP- 1 receptor activation has longer-term consequences, such as increased b cell proliferation and neogenesis along with cytoprotective (non-insulinotropic) effects [45–51].

### GLP- 1 Receptor Signaling

The heterotrimeric G proteins are known to mediate signaling through the canonical GLP- 1 receptor. These G proteins have both a Gb/c dimer subunit and an independent Ga subunit. GPCRs that are agonist-activated promote the synthesis of guanine triphosphate (GTP), which when bound to the G-protein causes the Ga and Gbc subunits to separate, which in turn can activate signaling proteins downstream [52]. It was initially discovered that GLP- 1 activates adenylate cyclase in tissue from the central nervous system (CNS) [53]. It has been demonstrated that ligand-activated GLP- 1 receptor interacts with the Gas subunit and activates adenylate cyclase to generate cAMP. Both the EPAC2 and PKA are activated in response to elevated cAMP [54]. By increasing Ca^2+^ influx and consequently insulin granule exocytosis, both pathways work together to promote insulin secretion. PKA directly phosphorylates sulfonylurea receptor 1 (SUR1), a regulatory subunit of voltage-dependent K^+^ channels and KATP channels, increasing membrane depolarization and voltage-gated Ca^2+^ channel activation. Through several mechanisms, including direct interaction with SUR1 and membrane depolarization, intracellular calcium mobilization, and insulin granule priming modification, EPAC2 activation may promote the exocytosis of insulin granules [55, 56].

There have also been reports of non-insulinotropic effects of GLP- 1 receptor activation. In fact, the GLP- 1 receptor-cAMP-PKA axis stimulates the growth of beta cells by activating the transducer of regulated cAMP response element-binding protein (CREB) (TORC2) and the expression of the CREB and insulin receptor substrate 2 genes [57] Additionally, by boosting bcl- 2 activity and blocking the proapoptotic Bax, activated CREB also supports beta cell survival [58]. GLP- 1's anti-apoptotic and proliferative effects are also mediated by the PI3 K/Akt axis through the transactivation of the epidermal growth factor receptor (EGFR), which is connected to GLP- 1 receptor through c-Src activation and the generation of endogenous EGF like ligands [59]. GLP- 1 receptor agonism has not been thoroughly studied, but it has been demonstrated in certain cell lines to activate additional G-protein subunits and alternative downstream signaling pathways [60–62]

### GIP Receptor Signaling

The mature 42 amino acid GIP attaches itself to the b cell surface and activates its cognate receptor, the GIP receptor. Ligand binding to the GIP receptor activates Gas, which in turn activates adenylate cyclase, resulting in the production of cAMP, in accordance with GLP- 1 receptor activation. PKA and EPAC are activated by elevated cAMP. Additionally, cAMP and PKA phosphorylate ERK1/2, which controls genes involved in proliferative and anti-apoptotic processes, and activate several proteins, such as the mitogen activated protein kinase (MAPK) cascades [63]. By the same mechanisms (potassium channel closure-mediated membrane depolarization) as the GLP- 1 receptor, the GIP receptor activation of PKA results in the secretion of insulin. Additionally, non-insulinotropic effects like regulating the survival and proliferation of pancreatic beta cells can be facilitated by GIP receptor activation [64, 65]. Furthermore, PKA activation is known to inhibit AMPK in GIP receptor and GLP- 1 receptor signaling, which results in the transfer of TORC2 into the nucleus [66]. The anti-apoptotic gene bcl- 2 is a promoter of transcription in the nucleus by a complex between CREB and TORC2. Additionally, the nuclear transcription factor (Foxo1) is phosphorylated upon activation of phosphoinositide- 3-kinase–protein kinase B/Akt (PI3 K-PKB/Akt), which in turn deactivates molecules to restrict the activity of proapoptotic pathways [66].

### Intracellular Signaling Pathways: Interaction and Cooperation in Beta Cells

The signaling pathways in beta cells that are triggered by ligand binding to the GIP receptor and GLP- 1 receptor share a lot of similarities. The PKA and EPAC2 pathways are triggered by both receptors'activation of adenylate cyclase, which raises intracellular cAMP and causes insulin synthesis and release, cellular proliferation, and antiapoptotic effects. Recruitment of beta arrestins desensitizes both receptors, and this is followed by internalization, recycling, and inactivation of Gas. There are also several distinctions. First, beta arrestins and Gaq can both internalize the GLP- 1 receptor, and multiple ligands can affect internalization. On the other hand, GIP receptor internalization depends solely on arrestins and is not easily impacted by new ligands [67]. By inhibiting the Gas proteins and facilitating receptor trafficking through internalization and recycling, these beta arrestins are essential for GIP receptor desensitization. Second, it has been demonstrated that GIP receptor activation in beta cells leads to MAPK-induced signaling pathways, whereas GLP- 1 receptor activation does not. Third, GIP receptor activation has not been shown to cause proliferation and anti-apoptosis; instead, GLP- 1 receptor activation stimulates EGF receptor signaling [20].

### GIP and GLP- 1 Signaling in Alpha Cells

**Fig. 1:**
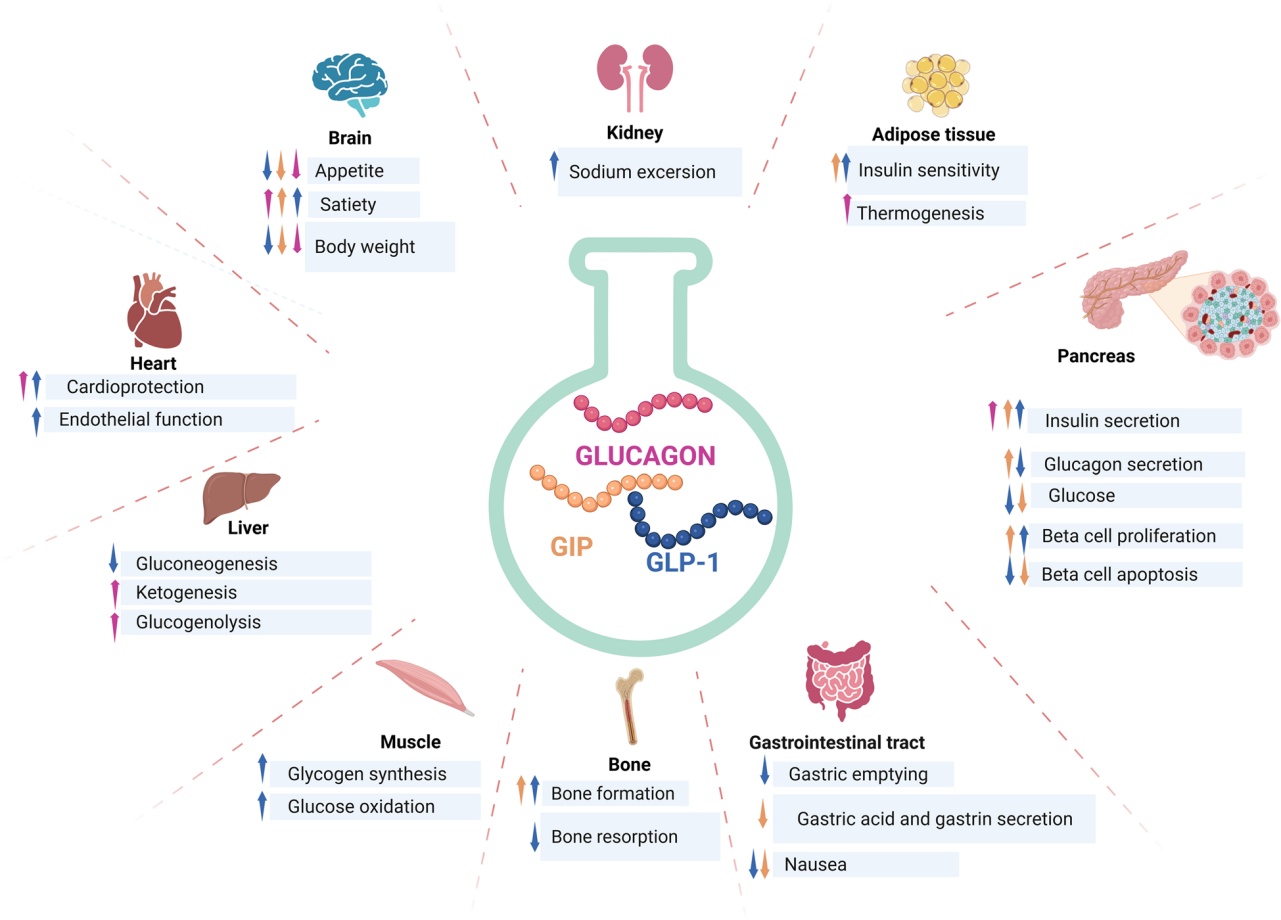
Role of GLP‐1, GIP and glucagon in different body organs. This shows the physiological effects of GLP‐1, GIP and glucagon on different organs and peripheral tissues. Created in BioRender. Anastasiou IA. (2025) https://BioRender.com/ a49a870. Assessed on 28 January 2025

It has been reported that GLP- 1 and GIP influence alpha cells. GLP- 1 stimulation also lowers blood sugar levels by inhibiting α cell production of glucagon [68]. A subset of cultured cells expressed GLP- 1 receptor, and mice with a-cell specific GLP- 1 receptor knockout have mild glucose intolerance and higher glucagon secretion in response to glucose challenges than animals of the wild type, which may indicate that GLP- 1 directly affects this cell [69]. It's also possible that GLP- 1 affects cells indirectly. In fact, it has been demonstrated that several molecules secreted by beta cells, such as insulin, zinc, and c-aminobutyric acid, inhibit the secretion of glucagon. These molecules may also theoretically help to inhibit the secretory activity of alpha cells that are dependent on GLP- 1 [70]. Furthermore, it is possible that somatostatin directly inhibits glucagon secretion by binding to its receptor in the alpha cell, since GLP- 1 stimulates islet somatostatin secretion directly through the canonical GLP- 1 receptor, expressed on delta cells [71]. Research on isolated perfused rat pancreas provides evidence in this regard. Treatment with anti-somatostatin antibodies or co-infusion with a somatostatin receptor 2 antagonist eliminated the GLP- 1-mediated suppression of glucagon secretion [71]. The direct action of GIP on its receptor, which is expressed by these cells, is what causes GIP stimulation to increase glucagon secretion by alpha cells in contrast to GLP- 1 [72–74]. Functionally, GIP receptor activation improves intracellular Ca^2+^ concentration, glucagon secretion, and cell depolarization by increasing cAMP/PKA signaling pathways [75, 76]. The intact rat pancreas's GIP perfusion only stimulates glucagon secretion at low glucose levels (4.4 mM) and not at postprandial glucose concentrations (8.9 mM), suggesting that GIP receptor activity in the cell appears to be glucose dependent [18]. In healthy humans, GIP-mediated elevation of circulating glucagon concentrations has also been observed; like in preclinical research, this effect is glucose dependent, occurring only in hypoglycemia [72, 77]. Figure 1 presents the main effects of GLP‐1, GIP and glucagon in different body organs.

### Preclinical data

In a randomized controlled trial, scientists reported the discovery and translational therapeutic effectiveness of a peptide with strong, balanced coagonism at both GLP- 1 and GIP receptors [78]. This unimolecular dual incretin, which was derived from a mixed sequence of GLP- 1 and GIP, showed improved antihyperglycemic and insulinotropic activity compared to selective GLP- 1 agonists. It is noteworthy that this superior effectiveness extended to primates (humans and cynomolgus monkeys) and rodent models of obesity and diabetes, such as db/db (diabetic) mice and Zucker diabetic fatty (ZDF) rat. In addition, a selective GIP agonist showed very low effectiveness in reducing weight, while this co-agonist showed synergism in reducing fat mass in obese rodents. The unimolecular dual incretins were more successful than selective monoagonists and tackled the two basic causes of diabetes: pancreatic insulin insufficiency and obesity-induced insulin resistance. To support less frequent administration, site-specific lipidation or PEGylation was performed to prolong the duration of action of the unimolecular dual incretins. These peptides offer pharmacology comparable to native peptides and improved effectiveness compared to similarly modified selective GLP- 1 agonists. The improvement in pharmacokinetics helped prevent the negative gastrointestinal side effects typical of GLP- 1-based selective agonists by reducing maximum drug exposure and reducing reliance on GLP- 1-mediated pharmacology. The identification and confirmation of a highly effective, balanced dual incretin agonist enables a more physiological approach to treating conditions associated with impaired glucose tolerance [78].

The combination of GLP- 1 and GIP is proposed as a promising therapeutic strategy for the treatment of obesity and T2DM. However, the neural mechanisms have not been fully elucidated. In a study, scientists investigated the role of GLP- 1 and GIP on central cells in regulating body weight homeostasis [79]. The effect of GLP- 1 together with GIP on food intake, body weight, and locomotor activity was determined following intracerebroventricular (ICV) administration of GLP- 1 and/or GIP in mice. ICV administration of low-dose GLP- 1 (0.3 nmol) and GIP (1 and 3 nmol) did not alter dietary intake. However, ICV administration of higher doses of GLP- 1 (1 and 3 nmol) and GIP (6 nmol) significantly reduced food intake and body weight. To investigate the synergistic effect of ICV-GLP- 1 and GIP, the subeffective dose GLP- 1 (0.3 nmol) and the subeffective dose GIP (1 nmol) were selected for further studies for concurrent administration. Concomitant ICV administration of GLP- 1 and GIP significantly reduced food intake, body weight, and alcohol consumption. Concurrent ICV administration of GLP- 1 and GIP significantly increased neuronal activation and pro-opiomelanocortin expression in the arcuate nucleus of the hypothalamus. Neural activation and POMC expressions were observed in two different neuronal populations. These results provide clues to neural mechanisms that support the development of GLP- 1 and GIP combination therapeutics for the treatment of obesity and DM [79].

Individuals with T2DM taking tirzepatide, a dual GIP and GLP- 1 receptor agonist, experienced better weight loss and blood sugar control than patients taking GLP- 1 receptor agonism. However, it is unclear how tirzepatide increases potency and what role GIP receptor agonism plays [80]. By increasing insulin sensitivity in obese mice more than GLP- 1 receptor agonism, researchers show that tirzepatide is an effective insulin sensitizer. The effect of tirzepatide was compared to wild-type obese and GLP- 1 receptor-null mice to determine whether GIP receptor agonism plays a role. By improving glucose removal in white adipose tissue, tirzepatide restored insulin sensitivity even when there was no GLP- 1 receptor -induced weight loss. The finding that a long-acting GIP receptor agonist (LAGIPRA) increases insulin sensitivity by promoting glucose capture in WAT confirms this. Interestingly, lower levels of branched-chain amino acids (BCAAs) and keto acids in the blood have been linked to the effect of tirzepatide and LAGIPRA on insulin sensitivity. The upregulation of genes associated with the breakdown of glucose, lipid, and BCAAs in brown adipose tissue has been linked to insulin sensitization. Overall, her research suggests that tirzepatide increased insulin sensitivity both independently and as a function of weight. These results shed light on the role that GIP receptor agonism plays in the therapeutic profile of dual receptor agonism and provide mechanistic explanations for the clinical effectiveness of tirzepatide [80].

In a study, researchers used a GIP receptor antagonist antibody (GIPRAb) and a GLP- 1 receptor agonist to target both pathways [81]. By creating GIP receptor -Ab/GLP- 1 bispecific molecules, they reduced body weight and improved numerous metabolic parameters in mice and monkeys, which is a strategy for treating obesity and its comorbidities. When GIPR-Ab/GLP- 1 is used instead of GIPR-Ab or a control antibody conjugate, the body weight loss is higher, indicating synergistic effects. Additionally, in diet-induced obesity (DIO) mice, GIP receptor-Ab/GLP- 1 lowers the respiratory exchange rate. Recombinant cells expressing both receptors produce more endosomal cAMP when GIPR-Ab/GLP- 1 binds to both receptors simultaneously and internalizes them quickly. This could be the reason why the bispecific molecules work so well. In general, GIPR-Ab/GLP- 1 molecules encourage body weight loss and have potential applications in the treatment of obesity [81].

In another study, researchers discovered that tirzepatide treatment, when combined with a decrease in circulating levels of branched-chain amino (BCAA) and keto (BCKA) acid metabolites linked to the development of systemic insulin resistance (IR) and T2DM improves insulin sensitivity in obese mice and humans with T2DM [82]. Crucially, it was discovered that these systemic effects were linked to elevated expression of BCAA catabolic genes in mice's thermogenic BAT. Tirzepatide may decrease circulating BCAAs/BCKAs by encouraging their catabolism in BAT, according to their hypothesis based on these findings. They used a mouse model of diet-induced obesity and combined metabolomic analyses in BAT and other tissues with stable-isotope tracer studies to answer this question. Through increased labeling of BCKA-derived metabolites and increases in levels of BCAA breakdown, such as glutamate, alanine, and 3-hydroxyisobutyric acid, they discovered that tirzepatide treatment stimulated catabolism of BCAAs/BCKAs in BAT. Additionally, tirzepatide administration over an extended period raised BAT levels of several amino acids that have been demonstrated to be elevated in response to cold exposure. Finally, long-term tirzepatide treatment significantly raised BAT levels of several TCA cycle intermediates, including fumarate, malate, and α-ketoglutarate. Lastly, they propose that tirzepatide causes a thermogenic-like amino acid profile in BAT, which could explain why obese IR mice have lower systemic BCAA levels [82].

In a study scientists evaluate the effects of glucagon, GIP, and GLP- 1 receptors in one compound (i.e. e. triagonism) on energy expenditure, glucose regulation, food intake, and weight loss in a well-established DIO mouse model in comparison to clinically relevant GLP1 receptor agonists [83]. Compared to GLP- 1 receptor mono-agonists and GLP- 1 receptor/GIP receptor co-agonists, optimized triagonists improve energy expenditure and restore body weight in DIO mice. These preclinical findings further implicate glucagon activation as the distinguishing factor between incretin receptor mono-, dual-, and triagonists, and they point to unimolecular polypharmacology as an efficient way to target several mechanisms causing obesity.

Table 1 includes preclinical studies of dual and triple use of GLP1 receptor co-agonists in T2DM and obesity [83].

**Table 1:** Preclinical studies of the dual and triple GLP1-RA use in T2DM and obesity

Authors, year, reference	Medication	Cells or animal model	Duration	Results
Finan et al., 2013 [78]	GLP- 1 and GIP receptors	C57BL/6 mice, db/db db/db (diabetic) mice, or ZDF rats	21 days	The identification and confirmation of a highly effective, balanced dual incretin agonist enables a more physiological approach to treating conditions associated with impaired glucose tolerance
NamKoong et al., 2017 [79]	GLP- 1 and GIP receptors	Male adult C57BL/6 J mice	1 h	The development of GLP- 1 and GIP combination therapeutics for the treatment of obesity and DM
Samms et al., 2021 [80]	Tirzepatide	WT, GLP- 1receptor –/–, and GIP receptor–/– mice on a C57BL/6 genetic background	14 days	GIP receptor agonism contributes to the therapeutic profile of dual receptor agonism, offering mechanistic insights into the clinical efficacy of tirzepatide
Lu et al., 2021 [81]	GIPR antagonist antibodies conjugated to GLP- 1	Cell lines; Normal mice; DIO mice; GIP receptor knockout mice; Normal cynomolgus monkeys; Obese cynomolgus monkeys	14 days	GIP receptor-Ab/GLP- 1 molecules contribute to body weight loss and have potential applications in the treatment of obesity
Samms et al., 2022 [82]	Tirzepatide	Mice	14 days	Tirzepatide induces a thermogenic-like amino acid profile in BAT, an effect that may account for reduced systemic levels of BCAAs in obese IR mice
Knerr et al., 2022 [83]	GLP- 1/GIP/glucagon triple agonists	Male C57BL/6 db/db mice	14 days	GLP- 1/GIP/glucagon triple agonists are an efficient way to target several mechanisms causing obesity

### Mechanism of GLP- 1 Action in T2DM

GLP- 1 increases insulin secretion through direct interaction with pancreatic beta cells. In addition, GLP- 1 reduces glucose production in the liver by preventing α-cells from secreting glucagon. It also indirectly increases insulin sensitivity in skeletal muscle by promoting microvessel recruitment, which may enhance local insulin action [69]. Numerous studies have also shown how important the portal glucose sensor is in maintaining glucose homeostasis and how GLP- 1 receptors are related to portal glucose sensing, with GLP- 1 being required for portal glucose to trigger the insulin response.

When GLP- 1 receptor agonists are used to treat T2DM, there is also a noticeable reduction in post-meal glycemic fluctuations due to their short-term, transient effect on slowing gastric emptying. The CNS GLP- 1 receptors are not necessary for the physiological regulation of blood glucose levels by endogenous GLP- 1 or the pharmacological control of glucose homeostasis that depends on GLP- 1 receptors, although further research is needed to investigate the possible role of GLP- 1 receptors expressed outside the beta cell, including within the enteric and peripheral nervous systems, in improving beta cell function through enteral glucose mediation [70, 84].

The incretin effect is reduced in people with T2DM, mainly because GIP has less of an impact [85]. Recent studies show that GLP- 1 function is impaired in people with T2DM, although GLP- 1 secretion is preserved. This can lead to reduced expression of GLP- 1 receptors and the development of GLP- 1 resistance. Other manifestations include decreased insulin secretion, increased insulin resistance and increased blood sugar levels [86].

Lastly, GLP- 1 receptor agonists'impact on weight may help improve blood glucose regulation and insulin sensitivity simultaneously.

### Commercially Available GLP- 1 Receptors Agonists for the Management of T2DM

#### Exenatide- Short Acting Agents

The first GLP- 1 receptor agonist used in clinical practice was exenatide, which was injected subcutaneously twice a day. It is an artificial peptide-based variant of exendin- 4, an incretin mimetic [87]. Exenatide is found in the saliva of the Gila monster and exhibits all the effects of naturally occurring GLP- 1 [87]. It decreased glycated hemoglobin (HbA1c) by − 0.78% at maximum dosages [88]. After being approved, daily lixisenatide, which is structurally like exenatide and is administered by subcutaneous injection—was discovered to lower HbA1c by 0.8 to 0.9% [89]. While lixisenatide is only available outside of the US, exenatide is commercially available twice daily in both Europe and the US. Lixisenatide and twice-daily exenatide were compared in the GetGoal-X trial, which found that lixisenatide was not inferior to exenatide in lowering HbA1c (− 0.79% vs. − 0.96%, in that order) [90].

#### Exenatide- Long-Acting Agents

Later, an extended-release formulation of exenatide was developed and given once a week. Exenatide administered once weekly as opposed to twice daily was found to lower HbA1c more significantly in the DURATION- 1 and DURATION- 5 trials (− 1.9% vs. − 1.5% and − 1.6% vs − 0.9%. correspondingly [90].

#### Liraglutide

Liraglutide, an analog of human GLP- 1, is given once daily by subcutaneous injection and is resistant to the inactivation by dipeptidyl peptidase- 4 (DPP- 4) [91]. In terms of lowering HbA1c, it proved to be more effective than exenatide twice daily, by − 1.12% vs. − 0.79% [90]. In addition, liraglutide reduced HbA1c to a greater extent compared to lixisenatide (− 1.8% vs. − 1.2%) [92].

#### Dulaglutide

Dulaglutide is another GLP- 1 synthetic analog which consists of two DPP- 4 resistant GLP- 1 molecules that are covalently linked to a modified IgG4 Fc fragment that acts as carrier, limiting the renal clearance of the molecule [93]. In terms of lowering HbA1c, dulaglutide, when administered once a week, was demonstrated to be more effective than exenatide twice a day, by − 1.51% vs.0.99% [94]. The AWARD- 6 trial revealed that dulaglutide was not inferior to liraglutide in terms of lowering HbA1c; − 1.42% vs. − 1.36%, in that order [95].

#### Semaglutide

The first GLP1-receptor agonist to be made available both orally and by subcutaneous injection was semaglutide. It shares structural similarities with liraglutide, but it has been modified to have a longer half-life and be even more resistant to DDP- 4 degradation. It has been demonstrated that semaglutide administered subcutaneously once weekly lowers HbA1c more effectively than liraglutide, dulaglutide, and exenatide once weekly [96]. In the PIONEER- 4 trial, semaglutide was not less effective than liraglutide at lowering HbA1c at its daily oral formulation dose [97]. But compared to dulaglutide, oral semaglutide significantly decreased HbA1c, by − 1.7 vs. 1.4% [97]. Comparative trials show that long-acting medications (exenatide once weekly, liraglutide, dulaglutide, and semaglutide) provide better glycemic control than short-acting medications (exenatide twice daily and lixisenatide) [90]. Semaglutide administered subcutaneously lowers HbA1c more than any other long-acting medication. Liraglutide and dulaglutide, as well as oral semaglutide and liraglutide, appear to offer comparable glycemic control. Although oral semaglutide might be a better option for patients who are allergic to injections or reluctant to self-inject, its efficacy may be limited by its stringent administration guidelines, which call for taking it half an hour before the first meal, drink, or other medication [90].

#### Dual Treatments

### Dual GLP- 1/GIP Receptor co-agonist – Tirzepatide

Tirzepatide is the first dual GLP- 1/GIP receptor co-agonist [98]. Glycemic control appears to be more effective when both receptors are activated [99, 100]. Greater effectiveness in blood glucose control appears to result from activation of both receptors. Compared to placebo, it has been shown to significantly lower HbA1c by up to 2.07% when administered as a once-weekly subcutaneous injection at a maximum dose of 15 mg. Compared to other single agonists, tirzepatide provides better glycemic control [99, 100].

### Dual GLP- 1 Receptor Agonists—CagriSema

A long-acting amylin analog called cagrilintide is being developed in conjunction with semaglutide 2.4 mg (CagriSema) to treat T2DM [101]. The injection is given subcutaneously once a week. In a phase 2 clinical trial, CagriSema reduced HbA1c by − 2.2% (p = 0.075) in comparison with semaglutide 2.4 mg alone [101].

### Dual GLP- 1 and Glucagon Receptor Agonists—Mazdutide and Survodutide

A subcutaneous injection of mazdutide is given once a week. In a Chinese phase II clinical trial, mazdutide decreased HbA1c by − 1.41% to − 1.67% in a dose-dependent manner, while a placebo decreased HbA1c by + 0.03% [102]. Survodutide was found to significantly lower HbA1c in comparison with a placebo in a phase II trial where it was administered subcutaneously once and twice a week [103].

### GLP- 1 Receptor Agonists in Development

Orforglipron is an oral, non-peptide, small molecule glucagon-like peptide- 1 receptor agonist [104]. Compared to peptide GLP- 1 receptor agonists, orforglipron, exhibits less receptor desensitization by increasing cyclic AMP signaling [105]. Oral orfoglipron, as opposed to peptide-based oral semaglutide, can be taken without regard to food timing. Orforglipron significantly reduced HbA1c to − 2.1% in a phase II clinical trial assessing its use in T2DM, while dulaglutide achieved − 1.1% and a placebo achieved − 0.43% [106]. In a phase II study, twice-daily danuglipron resulted in a dose-dependent reduction in HbA1c of − 0.49% to − 1.18%, while a placebo caused a decrease of − 0.02% [107].

### GLP- 1 Receptor Agonists in Combination with Insulin

The GLP- 1 receptor agonists have been shown to improve blood glucose control when used in conjunction with insulin, particularly basal insulin, and may be particularly useful in the treatment of advanced T2DM [108]. For example, the addition of semaglutide at a dose of 0.5 mg or 1 mg per week in patients receiving basal insulin resulted in a − 1% and − 1.8% decrease in HbA1c, respectively, in SUSTAIN- 5. The risk of weight gain and hypoglycemia can also be reduced with this combination therapy. Nevertheless, there is a higher risk of hypoglycemia than with single GLP- 1 treatment. The receptor agonist alone is less effective than insulin alone [108].

### Triple GLP- 1/GIP/Glucagon Receptor Agonists- Retatrutide

Retatrutide is being developed as a once-weekly subcutaneous injection of a triple GLP- 1, GIP, and glucagon receptor agonist to treat T2DM [109]. Retatrutide decreased HbA1c by − 2.02% in a phase II clinical trial, while dulaglutide 1.5 mg decreased HbA1c by − 1.14% and a placebo decreased it by − 0.01% [109]. Table 2 summarizes RCTs of dual and triple GLP1-RA use in T2DM.

**Table 2:** RCTs of the dual and triple GLP1-RA use in T2DM

Trial name or authors, year, reference	Medication	Inclusion criteria	Duration	Change in HbA1c
SURPASS- 1, Rosenstock et al., 2021 [100]	Dual (GLP- 1/GIP) Agonist tirzepatide 5 mg, tirzepatide 10 mg, tirzepatide 15 mg, placebo	Adults aged 18 and older who are taking metformin and/or lifestyle changes and have a BMI of at least 23 kg/m^2^ and a HbA1c of 7.0 to 9.5%	40 weeks	tirzepatide 5 mg = − 1.87 tirzepatide 10 mg = − 1.89 tirzepatide 15 mg = − 2.01 placebo = + 0.04
CagriSema, Frias et al., 2023 [101]	Dual (GLP- 1/GIP) agonist semaglutide/cagrilintide 2.4 mg, Semaglutide 2.4 mg, cagrilintide 2.4 mg	Adults aged 18 and older who were taking metformin with or without an SGLT- 2 inhibitor and had a BMI of at least 27 kg/m^2^ and a HbA1c of 7.5–10.0%	32 weeks	semaglutide/cagrilintide 2.4/2.4 mg = − 2.2 semaglutide 2.4 mg = − 1.8 cagrilintide 2.4 mg = − 0.9
Rosenstock et al., 2023 [109]	Triple (GLP- 1/GIP/glucagon) Agonist retatrutide 0.5 mg, retatrutide 4 mg (escalation from 2 mg), retatrutide 4 mg (no escalation) retatrutide 8 mg (slow escalation) retatrutide 8 mg weekly (fast escalation) retatrutide 12 mg, dulaglutide 1.5 mg, placebo = 45	People aged 18 to 75 who are on metformin, sulfonylurea, or lifestyle changes and have a BMI of 25 to 50 kg/m^2^ and a HbA1c of 7.0 0 to 10.5%	24 weeks	retatrutide 0.5 mg = − 0.43 retatrutide 4 mg (escalation from 2 mg) = − 1.39 retatrutide 4 mg (no escalation) = − 1.30 retatrutide 8 mg (slow escalation) = − 1.99 retatrutide 8 mg (fast escalation) = 1–0.88 retatrutide 12 mg = − 2.02 dulaglutide 1.5 mg = − 1.41 placebo = − 0.01

### Mechanism of Action of GLP- 1 in Obesity

The function of GLP- 1 in controlling feeding behavior and energy balance was investigated in rats. According to these studies, satiated rats eat more when exendin (9–39), a GLP- 1 receptor antagonist, is injected directly into their brains. Rats'appetites increase and their weight increases when the proglucagon gene, which encodes GLP- 1, is less expressed in the nucleus tractus solitarius [110, 111]. Administration of GLP- 1 receptor agonists reduces appetite, decreases energy intake, and increases satiety. According to functional magnetic resonance imaging, anticipatory food reward – the anticipated pleasure of eating certain meals – is reduced when GLP- 1 R is activated [110, 111].

### GLP- 1 Receptor Agonists for the Treatment of Obesity

#### Liraglutide

Liraglutide at a high dose of 3 mg daily is approved for long-term weight control in adults and children over the age of 12 with obesity or overweight plus at least one weight-related comorbidity. In adults with obesity, daily subcutaneous administration of 3.0 mg liraglutide in combination with lifestyle therapy results in a reduction in body weight of approximately 8%. This can increase to 11.5–15.7% when intensive behavioral therapy, exercise and calorie restriction are added [112]. Liraglutide helps prevent weight gain by reducing sedentary time and, when combined with exercise, improving cognitive control [112, 113]. It also counteracts the increased appetite that follows weight loss [112, 113]. Over 40 weeks, liraglutide 3.0 mg plus lifestyle modification reduced visceral adipose tissue in adults with overweight or obesity and at high risk of cardiovascular disease (excluding diabetes) compared to placebo (12.5% vs. 1.6%) [114]. Liraglutide 3.0 mg reduced BMI by 4.3% over 56 weeks in adolescents with obesity, while placebo reduced BMI by 0.3% in the long term [115]. Efficacy was predicted by early response to treatment [115].

#### Semaglutide

Subcutaneous semaglutide 2.4 mg once weekly is authorized for long-term weight control for adults and children over the age of 12 with obesity or overweight with at least one weight-related comorbidity [112]. In addition to aiding weight loss, semaglutide 2.4 mg also enhances eating regulation, lessens food cravings, and lessens the typical rise in appetite that follows substantial weight loss, all of which support continued weight control [112].

According to the Semaglutide Treatment Effect in People with Obesity (STEP) program studies, people with overweight or obesity (without T2DM) who received semaglutide 2.4 mg and underwent lifestyle interventions experienced an average weight reduction of 14.7- 17.4% [116]. These weight reductions were associated with improvements in cardiometabolic risk factors and sustained effects over two years. Individuals with overweight or obesity and T2DM were found to have a lower body weight reduction of 9.6%. Semaglutide 2.4 mg significantly decreased total body weight loss in individuals with obesity by 16.1% as opposed to 0.6% with a placebo. It also resulted in a 13.2% decrease in body weight in an East Asian population with overweight or obesity, with or without T2DM. After 68 weeks, weight loss was higher with one-weekly subcutaneous semaglutide 2.4 mg compared with once-daily liraglutide 3.0 mg (− 15.8% vs. − 6.4%). The need for treating obesity as a chronic condition is highlighted by the fact that weight regain happens after stopping semaglutide, despite lifestyle modifications [116].

### Injectable Dual Gut Peptide Agonists are Being Developed to Treat Obesity

#### Tirzepatide

Tirzepatide has recently been approved for long-term weight control in adults with obesity or overweight and at least one weight-related comorbidity. Compared to individual GLP- 1 receptor agonists, its action on GIP and GLP- 1 receptors improves appetite control and metabolic function, indicating higher efficacy [117]. Tirzepatide is given subcutaneously once a week and was examined in four worldwide phase III randomized, placebo-controlled clinical trials as part of the SURMOUNT Program, which also includes lifestyle modification. People with obesity or overweight (without T2DM) showed significant weight loss; 18.4–20.9%, with the 10 mg and 15 mg, along with improvements in cardiometabolic risk factors [118–120]. Tirzepatide doses of 10 mg and 15 mg resulted in lower body weight reductions of 12.8% and 14.7% respectively in individuals with overweight or obesity and T2DM [121].

#### Survodutide

In a 46-week randomized, double-blind phase II trial, researchers recruited people with overweight or obesity but not T2DM [122]. They found that at the highest dosage of 4.8 mg survodutide weekly, study participants lost 14.9% of their body weight in comparison with a placebo group (2.8% weight loss) [122].

#### Mazdutide

A phase II, randomized, double-blind, placebo-controlled trial showed moderate weight loss with mazdutide in Chinese patients with overweight or obesity without T2DM). Improved cardiometabolic risk factors vs placebo were observed after 24 weeks [123].

### Maridebart/cafraglutide (MariTide), an Antibody-peptide Conjugate Molecule

Maridebart/cafraglutide, or AMG 133, is a bispecific molecule that was created by conjugating two GLP- 1 analogue agonist peptides with a fully human monoclonal anti-human GIP receptor antagonist using amino acid linkers [124]. The ability of AMG 133 to lower body weight and improve metabolic markers in male obese mice and cynomolgus monkeys is reported by researchers, who also confirm the GIP receptor antagonist and GLP- 1 receptor agonist activities in cell-based systems. AMG 133 demonstrated significant dose-dependent weight loss in a phase 1 randomized, double-blind, placebo-controlled clinical trial involving participants with obesity, along with an acceptable safety and tolerability profile [124].

### Triple-Hormone-Receptor Agonist Retatrutide for Obesity

Weekly retatrutide injections (1 mg—12 mg) resulted in weight reductions of 7.2–17.5% at 24 weeks and 8.7–24.2% at 48 weeks in subjects with overweight or obesity (without T2DM) in a phase II, double-blind, randomized, placebo-controlled [125]. There was no plateau during this time [125]. Table 3 summarizes RCTs of the dual and triple GLP1-RA use in obesity.

**Table 3:** RCTs of dual and triple GLP1-RAs use in obesity

Trial name or authors, year, reference	Medication	Inclusion criteria	Duration	Weight change (%)
SURMOUNT- 1, Jastreboff et al., 2022 [118]	tirzepatide 5 mg, tirzepatide 10 mg, tirzepatide 15 mg, placebo	≥ 18-year-old adults without DM who have at least one failed weight loss attempt, a BMI of 30 kg/m^2^ or higher, and at least one weight-related condition	72 weeks	tirzepatide 5 mg = − 15.0% tirzepatide 10 mg = − 19.5% tirzepatide 15 mg = − 20.9% placebo = − 3.1%
SURMOUNT- 2, Garvey et al., 2023 [121]	tirzepatide 10 mg, tirzepatide 15 mg, placebo	Adults aged 18 and older who have a BMI of at least 27 kg/m^2^ and at least one weight-related condition, such as DM	72 weeks	tirzepatide 10 mg = − 12.8% tirzepatide 15 mg = − 14.7% placebo = − 3.2%
SURMOUNT- 3, Wadden et al., 2023 [120]	tirzepatide, placebo	≥ 18-year-old adults without DM who have at least one failed weight loss attempt, a BMI of 30 kg/m^2^ or higher, and at least one weight-related condition	84 weeks	72 weeks with tirzepatide: tirzepatide = − 18.4% placebo = + 2.5% 84 weeks (12 weeks of intensive lifestyle intervention + 72 weeks of tirzepatide or placebo): tirzepatide = − 24.3% placebo = − 4.5%
Ji et al., 2023 [123]	mazdutide 3.0 mg, mazdutide 4.5 mg, mazdutide 6.0 mg, placebo	Adults aged 18 to 75 who are overweight (BMI ≥ 24 kg/m^2^), have hyperphagia, at least one weight-related comorbidity, or obesity (BMI ≥ 28 kg/m2) but without DM	24 weeks	mazdutide 3.0 mg = − 6.7% mazdutide 4.5 mg = − 10.4% mazdutide 6.0 mg = − 11.3% placebo = − 1.0%
Jastreboff et al., 2023 [125]	retatrutide 1 mg, retatrutide 4 mg (initial dose 2 mg), retatrutide 4 mg (initial dose 4 mg), retatrutide 8 mg (initial dose 2 mg), Retatrutide 8 mg (initial dose 4 mg), retatrutide 12 mg (initial dose 2 mg), placebo	Adults aged 18 to 75 with at least one failed attempt at weight loss, a BMI of 30 to 50 kg/m^2^ or ≥ 27 kg/m^2^, at least one weight-related condition, and no DM	48 weeks	At week 24: retatrutide 1 mg = − 7.2% retatrutide 4 mg (initial dose 2 mg) = − 11.8% retatrutide 4 mg (initial dose 4 mg) = − 13.9% retatrutide 8 mg (initial dose 2 mg) = − 16.7% retatrutide 8 mg (initial dose 4 mg) = − 17.9% retatrutide 12 mg (initial dose 2 mg) = − 17.5% placebo = − 1.6% At week 48: retatrutide 1 mg = − 8.7% retatrutide 4 mg (initial dose 2 mg) = − 16.3% retatrutide 4 mg (initial dose 4 mg) = − 17.8% retatrutide 8 mg (initial dose 2 mg) = − 21.7% retatrutide 8 mg (initial dose 4 mg) = − 23.9% retatrutide 12 mg (initial dose 2 mg) = − 24.2% placebo = 2.1%
SURMOUNT- 4, Aronne et al., 2024 [119]	tirzepatide, placebo	≥ 18-year-old adults without DM who have at least one failed weight loss attempt, a BMI of 30 kg/m^2^ or higher, and at least one weight-related condition	88 weeks	At week 36 tirzepatide = − 20.9% From week 36–88 tirzepatide = − 5.5% placebo = + 14.0%
le Roux et al., 2024 [122]	survodutide 0.6 mg, survodutide 2.4 mg, survodutide 3.6 mg, survodutide 4.8 mg, placebo	Adults aged 18 to 75 without DM who have made at least one failed attempt to lose weight and have a BMI of 27 kg/m^2^ or higher	46 weeks	survodutide 0.6 mg = − 6.2 survodutide 2.4 mg = − 12.5 survodutide 3.6 mg = − 13.2% survodutide 4.8 mg = − 14.9% placebo = − 2.8%
Véniant et al., 2024 [124]	MariTide 140 mg, MariTide 280 mg, MariTide 420 mg, placebo	Individuals aged 18 to 65 without DM and BMI of 30 to 40 kg/m^2^	12 weeks	MariTide 140 mg = − 7.2% MariTide 280 mg = − 9.9% MariTide 420 mg = − 14.5% placebo = − 1.5%

### Obesity Treatment is Being Explored Through the Development of Oral Medications that Function as GLP- 1 Receptor Agonists

#### Semaglutide

A randomized, double-blind, phase III study found that giving adults overweight or obese (without T2DM) 50 mg of oral semaglutide once daily for 68 weeks, in addition to diet and exercise, significantly reduced their body weight in comparison with a placebo (15% vs. 2.4%) [126].

#### Orforglipron

**Fig. 2:**
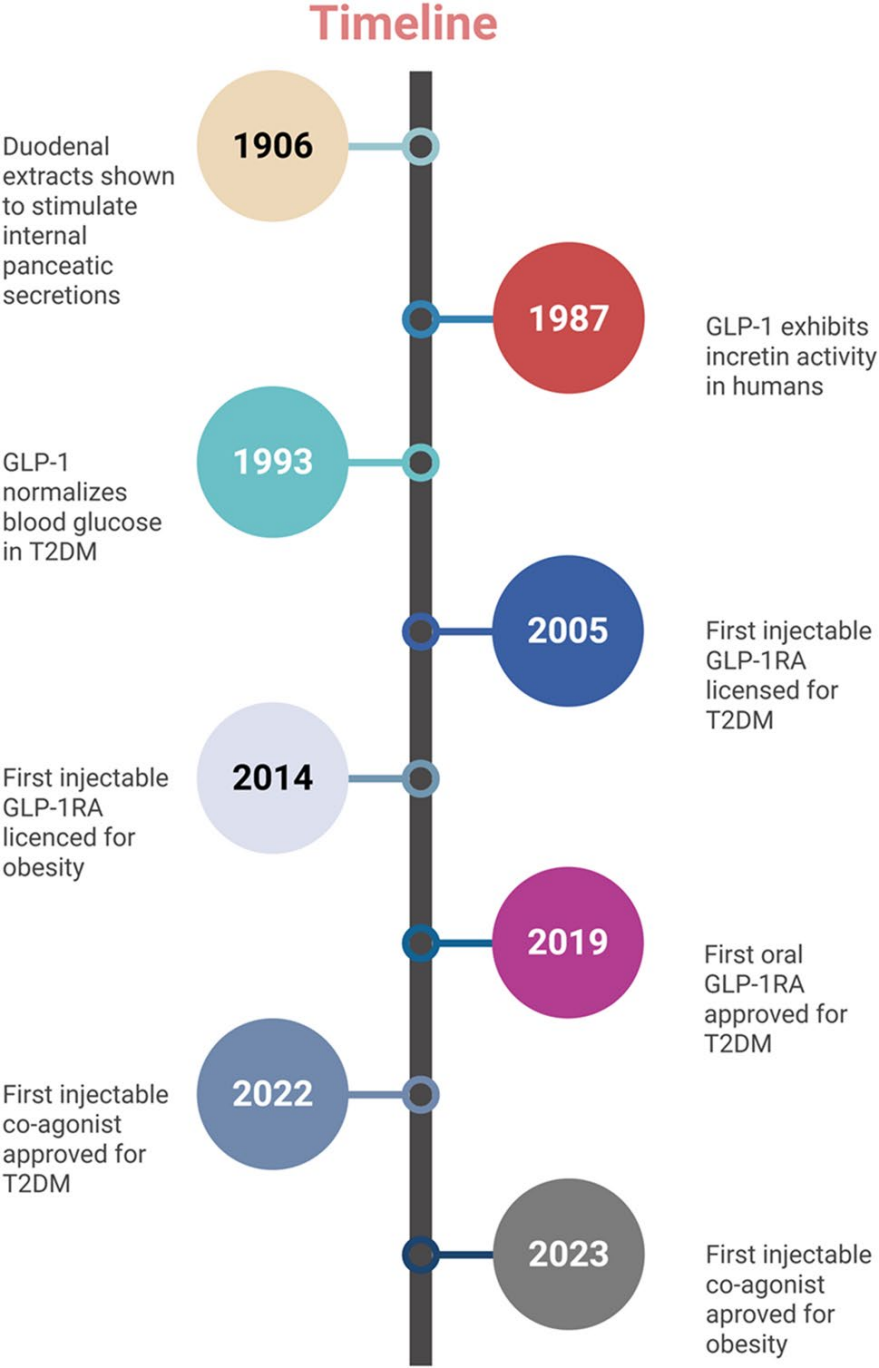
A timeline of GLP- 1 discovery and clinical development. Created in BioRender. Anastasiou IA. (2025) https://BioRender.com/s61k676. Assessed on 17 February 2025

In phase II, randomized, double-blind study, adults with overweight or obesity and without T2DM who took oral orforglipron (12, 24, 36, or 45 mg) daily for 36 weeks experienced a weight reduction of 9.4–14.7% [105]. Those who received a placebo experienced a weight loss of 2.3%, with no plateau observed. Orforglipron side effects were comparable to those of injectable GLP- 1 receptor agonists [105]. Figure 2 presents a timeline of GLP- 1 discovery and clinical development.

### Limitations of RCTs presented in Table 2 and Table 3

The side effects reported at the end of a clinical trial may be partially distorted due to gastrointestinal problems if the study is stopped too early [127]. The absence of validated assessment tools (e.g. a stable 13 C-labeled fatty acid breath test) and reliance on self-reported symptoms rather than validated symptom indices for gastrointestinal disorders are also limitations. In addition, differences in the initial situation of gastric emptying, which is a measure of the effectiveness of GLP- 1- receptor co-agonists, are likely to have a different effect on patients with obesity than on people in whom preoperative intragastric food retention after taking GLP- 1 receptor co-agonists is not impaired, and in people with diabetes [127]. Although the American Society of Anesthesiologists Working Group on Preoperative Fasting is based primarily on anecdotal reports and case reports, it has issued guidelines for taking these medications before non-urgent or urgent procedures that require general anesthesia due to concerns about the risk of regurgitation and lung aspiration due to delayed gastric emptying [128]. The risk of biliary disease has been examined in fewer studies, which may be because gallbladder emptying is impaired. Nevertheless, tirzepatide use seemed to be associated with a higher risk (RR 1.97, 95% CI 1.14–3.42, p = 0.558) [129]. Both liraglutide and exenatide showed significant decreases in liver fat, liver enzymes, and fibrosis index associated with metabolically associated steatotic liver disease (MASLD) [130]. In contrast, compared to placebo, semaglutide resulted in a 59% improvement in MASLD and an improvement in liver fibrosis, which slowed the progression of the disease [130]. According to other recent analyses, tirzepatide, retatrutide, and survodutide all significantly reduce liver fat without showing signs of worsening liver fibrosis markers [131].

### Α comparative Analysis of Bariatric Surgery with Dual and Triple GLP- 1 Receptor Agonists

The goal of treating obesity is to reduce weight over time while improving health and quality of life [132, 133]. Although lifestyle changes are the first line of treatment for obesity, even the most rigorous lifestyle changes typically only produce a 5–10% weight loss and maintaining weight over time is difficult due to compensatory physiological processes that increase appetite and decrease energy expenditure [134]. To improve or achieve remission of some obesity-related complications and lower risk for cardiovascular events, individuals with a BMI of ≥ 35 kg/m^2^ may need to lose more weight (10–25%). 5–10% weight loss is considered clinically meaningful with pharmacotherapy. Bariatric surgery for qualified individuals is the next option if lifestyle changes and pharmacotherapy are unable to meet individualized weight loss goals [132]. Although bariatric surgery can lead to weight loss of at least 25% and long-term weight maintenance, it is not scalable at the population level, and many people are still afraid to have surgery because of the possibility of complications, [135, 136].

A better understanding of the complex physiology of weight regulation and the role of intestinal hormones in regulating food intake, appetite, and glucose homeostasis led to the discovery of GLP- 1 receptor analogs as safe and effective treatments, initially for T2DM and more recently in higher doses to treat obesity (liraglutide 3 mg and semaglutide 2.4 mg). Semaglutide 2.4 mg weekly, a novel GLP- 1 receptor agonist, was the first approved treatment for obesity. In 2021, it resulted in a mean weight loss of roughly 15% and maintenance through decreased appetite. Semaglutide 2.4 mg once weekly may not work well for certain populations, and the weight loss from bariatric surgery varies greatly (32% of patients with T2DM achieve ≤ 5% weight loss with semaglutide 2.4 mg) [112]. Furthermore, some patients cannot tolerate injectable treatments, and GLP- 1 receptor agonist side effects typically depend on it. Despite the lack of extra support for lifestyle modification as part of the SURPASS program, 40–69% of patients achieved ≥ 10% weight loss with the highest doses of tirzepatide (10–15 mg), which ranged from 9.5 kg to 12.9 kg [100].

**Fig. 3:**
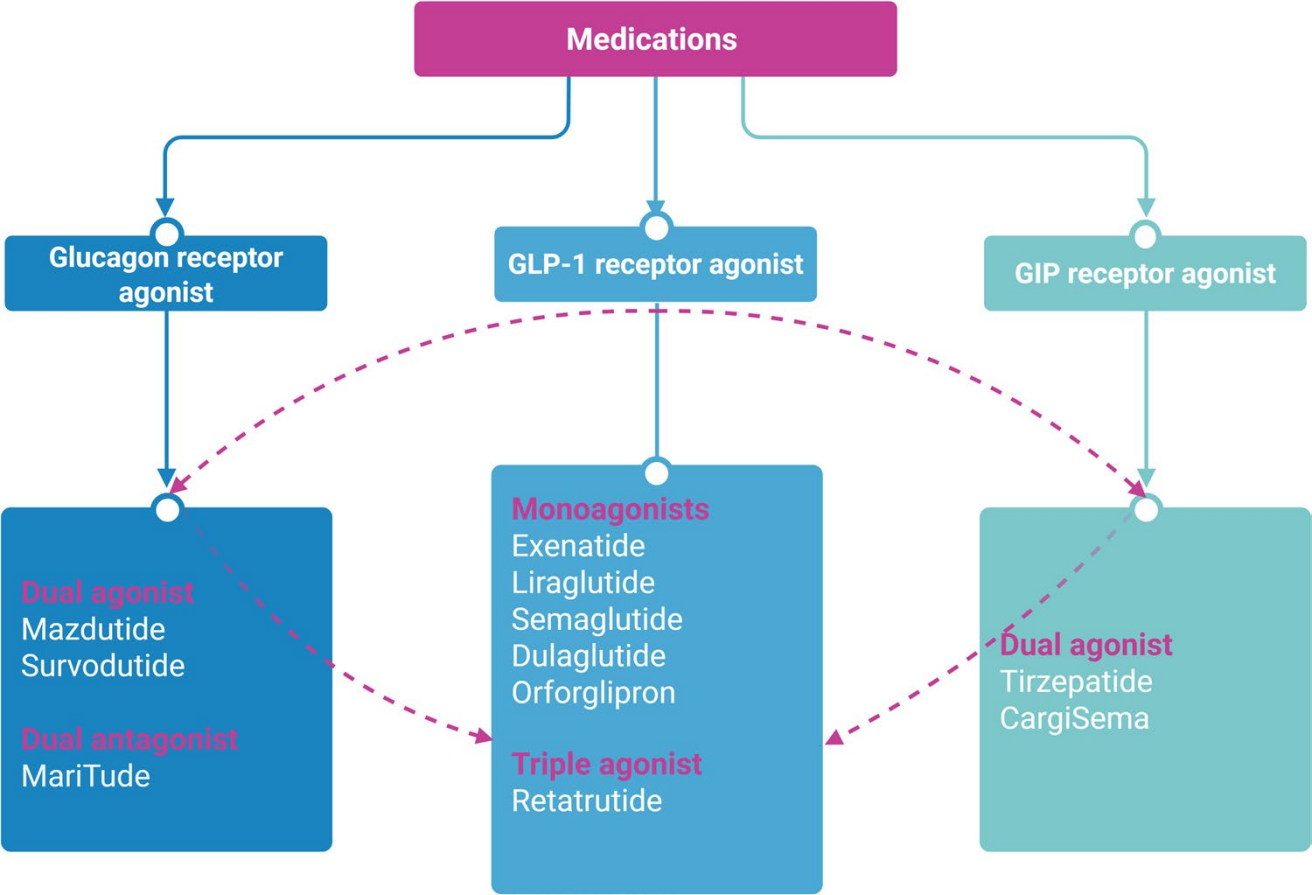
Schematic representation of monoagonists, dual agonists, antagonists and triple agonists based on activation of GLP- 1, GIP and glucagon receptor alone or in combination. Created in BioRender. Anastasiou IA. (2025) https://BioRender.com/q97u579. Assessed on 28 January 2025

In individuals with obesity without T2DM, SURMOUNT- 1 evaluated the safety and effectiveness of tirzepatide 5, 10, and 15 mg once weekly in comparison to a placebo when paired with a 500 kcal/day deficit diet and recommendations for 150 min of weekly exercise [118]. The average weight loss after 72 weeks of treatment was 15–20% (compared to 3.1% with a placebo), and 30–57% of participants lost at least 20% of their body weight [118]. Retatrutide administered once weekly subcutaneously for 12 weeks resulted in a dose-dependent placebo-adjusted weight loss of up to 8.96 kg in the highest dose group and a placebo-adjusted HbA1c reduction of up to 1.6% in a phase II trial in individuals with T2DM [125]. It seems that pharmacotherapy is starting to catch up with bariatric surgery with regards to initial weight loss, but long-term data are still not available. The combination of different treatment modalities with complementary measures is the standard of care for many chronic and complex diseases. It is becoming increasingly obvious that treating obesity also requires a multimodal approach that combines lifestyle interventions, pharmacotherapy, and bariatric surgery to achieve the tailored weight loss and metabolism goals needed for long-term health gain. This is because weight gain is common even after bariatric surgery and can lead to the recurrence of obesity-related complications. Combination therapies should be offered as a first line of treatment and not as a last resort for patients with severe and complex obesity. It is therefore important to question the traditional step-by-step approach to treating obesity and to tailor treatments to individual patients'needs. The safety and effectiveness of recently developed and future expected obesity drugs combined with other treatment modalities (such as bariatric surgery or drastic lifestyle changes) need to be further investigated. [132]. Thus, following the example of cancer, drug therapy could precede bariatric surgery in order to render it safer and more feasible, but could also follow if the results are not satisfactory. Figure 3 presents medications based on monoagonists, dual agonists, antagonists and triple agonists.

### Further Hormone Combinatorial Approaches with Promising Data

Other combination pharmacotherapies based on gut hormones have shown positive effects in preclinical models. Associating GLP- 1 or glucagon receptor agonists with core hormones like estrogens, thyroid hormones, and dexamethasone was the strategy employed (reviewed in [137, 138]). For example, a GLP- 1/oestradiol conjugate reduced feeding and body weight in several rodent models of the metabolic syndrome [139]. GLP- 1/oestradiol conjugate was more effective than GLP- 1 alone and triggered the insulin signaling pathway in beta cells, which led to remission of DM and restoration of beta cell function [140]. Thyroid hormones were delivered to the liver specifically by conjugating T3 with glucagon; in multiple mouse models of obesity, this led to a decrease in body weight and the correction of dyslipidemia [141]. Finally, obese mice lost weight more effectively when dexamethasone and GLP- 1 were conjugated than when GLP- 1 was used alone [142]. Combination pharmacotherapy, which involves not only gut hormones but also other molecules, appears to be a viable treatment option for metabolic syndrome. Due to their preclinical testing and lack of comparison with other dual receptor agonists such as tirzepatide, further studies are needed to determine whether the effects of these unimolecular compounds are superior to those of clinically tested drugs.

### Side Effects of GLP- 1 Medications

The effectiveness of GLP- 1 and dual GLP- 1/GIP receptor agonists in the treatment of obesity and overweight has been thoroughly established [143]. As with any medicine, there is a possibility of unwanted effects. Most side effects of GLP- 1 and GLP- 1/GIP receptor agonists are mild to moderate and resolve over time. These are usually gastrointestinal complaints. Concerns have been raised about serious side effects (e.g., gastrointestinal paralysis). The concerns regarding the possibility of medullary thyroid cancer, pancreatic cancer, suicidal ideation have been allayed by recent data. What is critical is that the side effect profiles of these medications are generally very acceptable. Obesity is one of the most common diseases worldwide. A significant risk of cardiovascular disease, cardiovascular death, and cardiometabolic risk factors is also associated with obesity. The same applies to cancers such as breast, ovarian, uterine, pancreatic, and esophageal cancer, among others. The benefits of these medications outweigh the risks for the majority of adults with overweight or obesity, as cancer and cardiovascular disease are the two leading causes of death for adults worldwide [143].

For some people, the benefits of these medications do not outweigh the risks, as with any other pharmacologic intervention (e.g., adults who have previously had medullary thyroid cancer, in which they are contraindicated). A comprehensive physical examination and detailed medical history should be performed to identify these individuals. The most important thing is that these medications are taken under the guidance of a doctor who is trained and experienced in their use. This allows patients to receive comprehensive information about the effectiveness of these medications, as well as advice about possible side effects. Shared decision-making should take place, considering their current health status. The aim is to offer efficient treatment to adults suffering from obesity, considering their individual characteristics, preferences and living conditions.

### Cost-effectiveness of GLP- 1 Medications

The demand for the significant weight loss that GLP- 1 receptor agonists and dual and triple anti-obesity agonists produce, the high cost of production, as well as their novelty, are serious factors which increase their price. Semaglutide was the most cost-effective treatment compared to exenatide, dulaglutide, liraglutide, or no treatment in short-term analyses (approximately one and a half years) that assessed quality-adjusted life years and incremental cost-effectiveness ratios [135–144]. However, compared to endoscopic sleeve gastroplasty, semaglutide is less cost-effective over 5 years with the same outcome parameters and a current annual price of approximately $13,618 in the US [144, 145].

### Unanswered Questions Regarding Weight Loss Mechanisms: Perspectives to Preclinical Research

The exact mechanisms underlying the weight loss are still not clear, especially in light of the enhanced effects seen with multi-agonism, despite the large number of incretin agonists presently undergoing clinical trials and their remarkable effectiveness in managing obesity and T2DM [5, 14, 146, 147]. Each incretin receptor agonist effects on body weight and energy metabolism may be slightly different and, in the case of dual and triple agonists, frequently complementary.

Incretin agonist clinical trials most frequently reveal nausea as an adverse effect. Future generations of incretin agonists may be able to maintain the reduction in blood glucose and body weight while minimizing these off-target effects. In preclinical models, GIP receptor agonism attenuates emesis while increasing the amount of body weight reduction in comparison to GLP- 1 agonism alone [148]. Although dual and triagonist medications have so far exhibited side effects comparable to those of GLP- 1 receptor monoagonists in human trials, these preclinical findings imply that GIP receptor agonism may be the secret to obtaining the weight loss advantages of incretin agonism with less severe side effects. Thus, comprehending the mechanisms underlying GIP receptor agonism is of particular interest. To date, we have learned that tirzepatide mainly stimulates insulin secretion through GIP receptor in human islets [149]. Also, the hypothalamus's GIP receptor signaling controls body weight and appetite [150]. Due to its CNS action, the dual GLP- 1/GIP dual agonist exhibits these superior benefits [23].

The main effect of endogenous GIP regarding weight is primarily on adipose tissue, where it promotes positive energy balance. It is probably broken down before it can reach CNS GIP receptors. The GIP-induced peripheral promotion of positive energy balance is likely to be far less effective than the GLP- 1/GIP dual agonist's direct action on the central nervous system, which results in satiety and weight loss. What parts of the brain and what kinds of cells are involved is another important question that has not been fully addressed.

Numerous cell types, including endothelial cells, tanycytes, and neurons in the hypothalamus and hindbrain express GLP- 1, GIP receptors and glucagon receptor, and. In a recent study, Liskiewicz and colleagues discovered that long-acting GIP receptor agonists activate GABAergic neurons that express the GIP receptor in the area postrema, and that GIP receptor signaling in GABAergic neurons is necessary for the effects of GIP to lower body weight [151].

## Conclusion

More than thirty years have passed since the gut hormone GLP- 1 was first shown to lower blood sugar levels in people with T2DM [152]. GLP- 1 receptor agonists have a mechanism of action that provides a multimodal strategy for the treatment of obesity and T2DM. GLP- 1 receptor agonists are extremely effective in controlling blood glucose levels because they increase insulin secretion, improve insulin sensitivity, and reduce glucose production in the liver. In addition, they support weight loss and control through their effect on satiety and appetite suppression. From short-acting to long-acting formulations, the wide range of GLP- 1 receptor agonists available provides physicians with numerous options to tailor treatment to each patient's needs.

In the treatment of obesity, GLP- 1 receptor agonists have shown remarkable efficacy in promoting weight loss and improving cardiometabolic parameters. When used alone or in conjunction with lifestyle changes, ligarglutide, semaglutide, tirzepatide, orforglipron, retatrutide and other newly developed drugs have demonstrated significant weight loss. In addition, new drugs such as tirzepatide have come onto the market, expanding the therapeutic options for successful treatment of T2DM and obesity. For patients with T2DM and obesity, GLP- 1 receptor agonists offer hope for better outcomes and a higher quality of life.

Despite their effectiveness, concerns remain regarding safety, tolerability and potential health risks, as well as the need for long-term administration and the subsequent cost. Nausea and diarrhea were common gastrointestinal side effects that occasionally caused people to stop taking the medication. Although rare, serious side effects have been noted, with gallbladder disease and pancreatitis among the most concerning. However, given the associated risks of cardiovascular disease and various cancers, the benefits of these medications, such as better glycemic control, weight loss and improvement in cardiometabolic risk factors as well as other obesity complications, appear to outweigh the risks for most adults with obesity [143, 153]. To balance risks and benefits, expert opinions emphasize the importance of individualized dosing, shared decision-making, and careful patient selection. Further research is needed to identify and manage possible side effects, such as the effects on gastric emptying and the need for safety precautions during anesthesia. Evidence-based recommendations are critical to ensure the safe and efficient use of these medications in the treatment of obesity as their use continues to increase.

Clinical efficacy studies of various unimolecular dual and triple agonists targeting the GLP- 1 receptor, GIP receptor, and glucagon receptor have shown promising results when used alone or in conjunction with approved hypoglycemic medications. The simultaneous activation of these G protein receptor signaling pathways on the islet cells may lead to additional antagonistic or synergistic interactions, but this is unknown. Before their cellular effects can be fully explained, more thorough studies are required to fully understand the signaling pathways affected by dual- and triagonism. To understand the long-term safety and chronic effectiveness of these treatments, these studies will be essential. Clinical decision support, management, risk detection, prevention, prognosis and diabetes diagnosis (including medical image analysis and subtype classification) are expected to be increasingly dependent on artificial intelligence [154–158].

## Key References

- Blüher M, Rosenstock J, Hoefler J, et al. Dose–response effects on HbA(1c) and bodyweight reduction of survodutide, a dual glucagon/GLP- 1 receptor agonist, compared with placebo and open-label semaglutide in people with type 2 diabetes: a randomised clinical trial. Diabetologia. 2024;67(3):470–482. 10.1007/s00125-023-06053-9.
  - A research article about the dose–response effects of survodutide, a dual agonist of the subcutaneous glucagon receptor and glucagon-like peptide- 1 receptor, on bodyweight loss and HbA1c levels.
- Wharton S, Blevins T, Connery L, et al. Daily Oral GLP- 1 Receptor Agonist Orforglipron for Adults with Obesity. N Engl J Med. 2023;389(10):877–888. 10.1056/NEJMoa2302392.
  - A research article on the effectiveness and safety of the non-peptide glucagon-like peptide- 1 (GLP- 1) receptor agonist Orforlipron than once daily oral therapy for weight reduction in adults with obesity.
- Aronne LJ, Sattar N, Horn DB, et al. Continued Treatment With Tirzepatide for Maintenance of Weight Reduction in Adults With Obesity: The SURMOUNT- 4 Randomized Clinical Trial. JAMA. 2024;331(1):38–48. 10.1001/jama.2023.24945.
  - A research article on evaluating the impact of tirepatide with nutrition and physical activity to maintain weight reduction.
- le Roux CW, Steen O, Lucas KJ, et al. Glucagon and GLP- 1 receptor dual agonist survodutide for obesity: a randomised, double-blind, placebo-controlled, dose-finding phase 2 trial. Lancet Diabetes Endocrinol. 2024;12(3):162–173. 10.1016/S2213-8587(23)00356-X.
  - A research article on the safety, compatibility and effectiveness of the glucagon receptor-GLP- 1 receptor dual agonist Survodutide in obesity management.

## Data Availability

No datasets were generated or analysed during the current study.

## Abbreviations

AC: Adenylate cyclase
ATP: ΑDenosine triphosphate
BAT: Brown adipose tissue
BCAAs: Branched-chain amino acids
BCKA: Branched-chain keto acids
BMI: Body mass index
cAMP: Cyclic adenosine monophosphate
CNS: Central nervous system
CREB: CAMP response element-binding
DIO: Diet-induced obesity
DPP- 4: Dipeptidyl peptidase- 4
EGFR: Epidermal growth factor receptor
EPACs: Exchange proteins directly activated by Camp
Foxo1: Nuclear transcription factor
GIP: Glucose-dependent insulinotropic polypeptide
GIPRAb: GIP receptor antagonist antibody
GPCRs: G-protein-coupled receptors
GTP: Guanine triphosphate
HbA1c: Glycated haemoglobin
HSL: Hormone-sensitive lipase
ICV: Intracerebroventricular
IR: Insulin resistance
LAGIPRA: Long-acting GIP receptor agonist
MAPK: Mitogen activated protein kinase
MASLD: Metabolically associated steatotic liver disease
PI3 K-PKB/Akt: Phosphoinositide- 3-kinase–protein kinase B/Akt
PKA: Protein kinase A
SUR1: Sulfonylurea receptor 1
T2DM: Type 2 diabetes mellitus
TORCs: Transducers of regulated CREB protein activity
ZDF: Zucker diabetic fatty
